# Regulation of shoot-system development in *Populus*

**DOI:** 10.1186/1753-6561-5-S7-I11

**Published:** 2011-09-13

**Authors:** Amy Brunner, Xiaoyan Sheng, Joesph Edwards, Takeshi Fujino, Chieh-Ting Wang, Stephen DiFazio

**Affiliations:** 1 Department of Forest Resources and Environmental Conservation, Virginia Tech, Blacksburg, VA 24061, USA; 2 Department of Biology, West Virginia University, Morgantown, WV 26506, USA

## 

Shoot phenology interacts with crown morphology to determine leaf production and duration over the growing season and throughout a tree’s life span; and thus, this interaction has a major role in determining whole-tree photosynthesis and biomass yield. For several years, the pattern of shoot meristem activity determines *Populus* crown architecture, but after the first onset of flowering the specification of meristem identity (inflorescence vs. vegetative) also has a major influence on crown form. To study how shoot meristem activity and identity are regulated in *Populus*, we are focusing on two pathways that have been extensively characterized in annual plants: 1) the *MORE AXILLARY BRANCHING* (*MAX*) genes that are involved in the synthesis and perception of upwardly mobile strigolactones that inhibit axillary bud outgrowth, and 2) members of the *TERMINAL FLOWER1* (*TFL1*)*/FLOWERING LOCUS T* (*FT*) and *APETALA1*(*AP1*)*/FRUITFULL*(*FUL*) gene families that regulate flowering. We are taking a multi-pronged approach that includes field and controlled-environment study of poplar transgenics, transcriptome analyses, cell-specific expression studies, and QTL and association mapping.

QTL mapping in a hybrid poplar pedigree localized three of seven poplar *MAX* genes in QTL for bud set and two *MAX* genes in sylleptic branching QTL. All *MAX* transgenics were established in a field trial and phenotyping of phenology, crown morphology, and growth is in progress. Greenhouse studies of poplar transgenics with the seven different poplar *MAX* gene promoters driving the reporter gene GUS show that most of the *MAX* genes are expressed in vascular tissues. However, vascular expression patterns are different, especially in stems undergoing secondary growth. Surprisingly, none of the RNAi transgenics showed differences in branching in the greenhouse under standard growing conditions. High nitrogen levels can induce sylleptic branching in poplar and greenhouse experiments with *MAX* transgenics indicate a possible role for one *MAX* gene in nitrogen-induced sylleptic branching. Ongoing studies of the effects of decapitation of the shoot apex with and without subsequent auxin application to the cut stem indicate that, as predicted based on studies in annual plants, the poplar *MAX* RNAi transgenics show a reduced response to apically-applied auxin.

Recent work has revealed that poplar *FT1* and *FT2* have diverged in regulation and function with *FT1* promoting the transition to flowering in response to winter temperatures, whereas warm temperatures and long days promote *FT2* expression and shoot growth [1]. In Arabidopsis, *FT* activates *AP1*, a key regulator of flower initiation. Expression and transgenic studies in poplar indicate that similar to the two poplar *FTs*, poplar *AP1* subfamily members have roles in both flowering and bud set in response to short-days and abiotic stress. Ongoing work is aimed at defining the roles of *FT* and *AP1* modules in these processes and to delineate the roles of the five different poplar *AP1/FUL* genes. Finally, *TFL1* acts opposite to *FT* to promote vegetative meristem identity in Arabidopsis, and *AP1* and *TFL1* repress each other’s expression. The poplar *TFL1* homologs, *CEN1* and/or *CEN2* have roles in maintaining vegetative identity and in the dormancy-growth transition [2] and we are currently working to define the individual roles of the poplar *CENs* and their relationships to *FT* and *AP1* activities.
